# Supplementary material for: Active tactile discrimination is coupled with and modulated by the cardiac cycle
Source: eLife. 2022 Oct 12;11:e78126. doi: 10.7554/eLife.78126 (PMC9671494; doi:10.7554/eLife.78126)
Supplement: Supplementary file 1. [file elife-78126-supp1.docx]

Supplementary file

**Active tactile discrimination is coupled with and modulated by the cardiac cycle**

Alejandro Galvez-Pol^1,2^*, Pavandeep Virdee^1^, Javier Villacampa^2^, James M Kilner^1^

**Supplementary File 1A. Post Hoc comparisons - Proportion correct responses by gratings difficulty (1-7)**


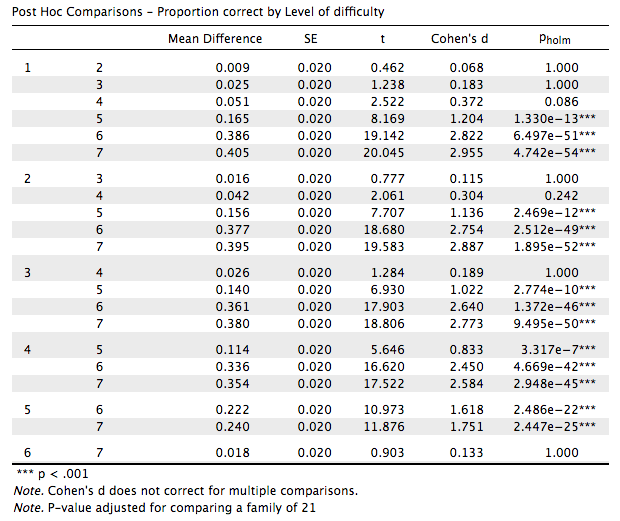


**Supplementary File 1B. - Proportion Mdn holding times by gratings difficulty (1-7)**


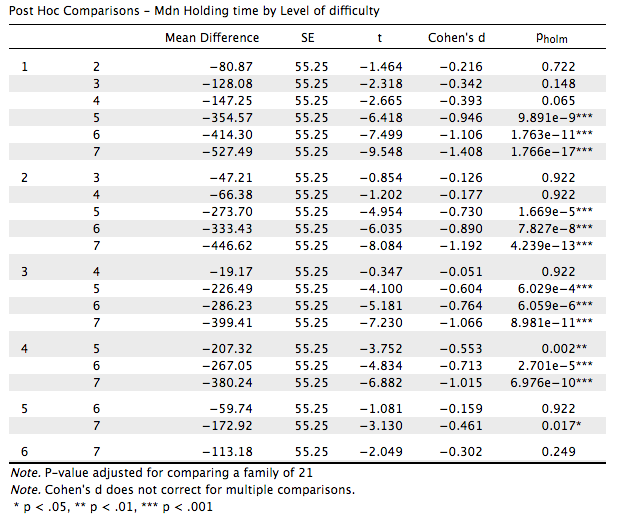


**Supplementary File 1C. Post Hoc tests Duration IBIs by heartbeat position (relative to touch entailing heartbeat). Gratings stimulus**

**
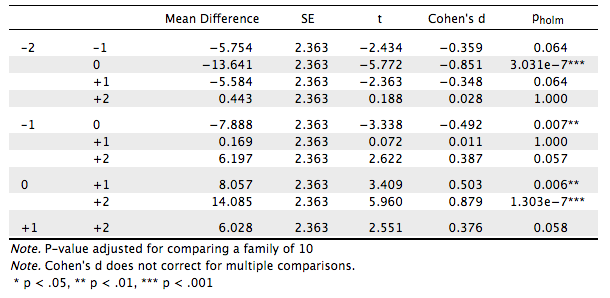
**

**Supplementary File 1D. Post Hoc tests Duration IBIs by heartbeat position (relative to touch entailing heartbeat). Flat control stimulus**

**
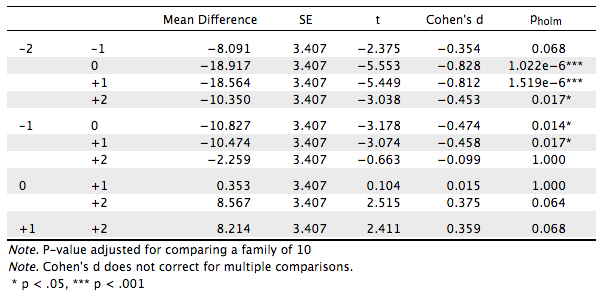
**

**Supplementary File 1E. Post Hoc tests Duration diastole by heartbeat position. Gratings stimulus**


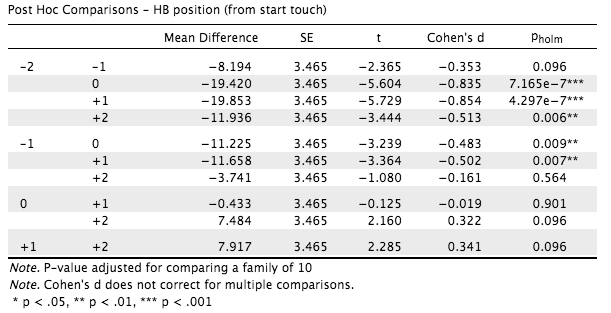


**Supplementary File 1F. Post Hoc tests Duration diastole by heartbeat position. Flat control stimulus**


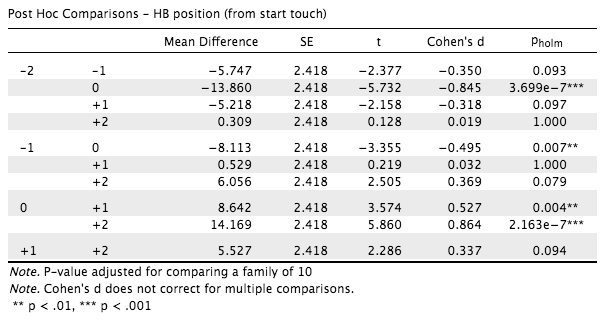


**Supplementary File 1G. Pearson’s r correlation between subject’s touch variability and proportion of correct responses (across whole experiment with all difficulty levels)**

**
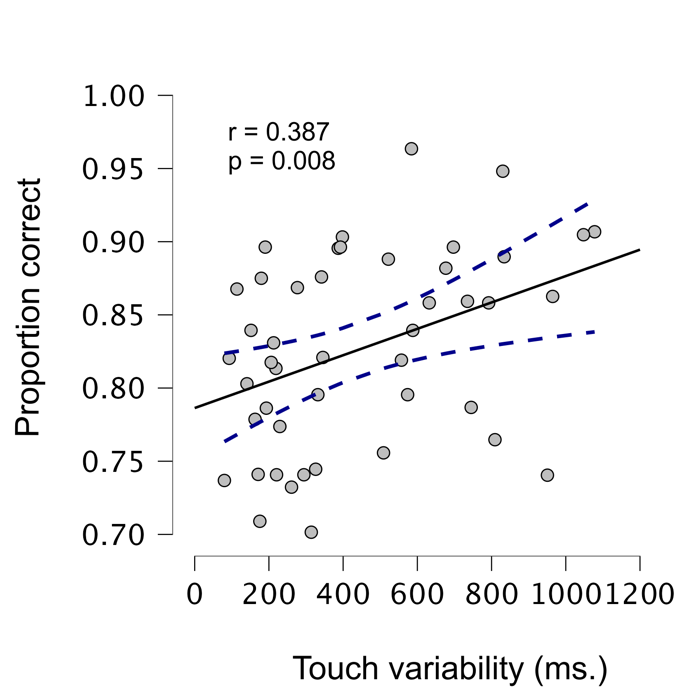
**

**Supplementary File 1H. Mdn holding times by cardiac phase x level of difficulty * heartbeat deacceleration**


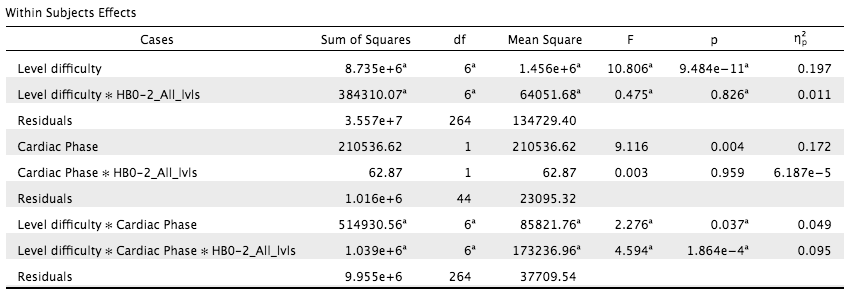


*Note*. Duration of subjects’ touch as a function of the cardiac phase in which it was initiated interacted significantly with task difficulty (Level of difficulty) when adding the covariate heartbeat deacceleration (i.e., HB0-2_All_lvls). Heartbeat deacceleration was computed by subtracting the duration of the heartbeat entailing the starting touch minus that of the second heartbeat before the starting touch (i.e., IBI entailing the touch in ms minus IBI of the second heartbeat before touch). Level of difficulty x Cardiac Phase * HBO-2_All_lvls, *p* = 0.0004.
